# Excess mortality in Denmark, Finland, Norway and Sweden during the COVID-19 pandemic 2020–2022

**DOI:** 10.1093/eurpub/ckae091

**Published:** 2024-05-17

**Authors:** Ingeborg Forthun, Christian Madsen, Louise Emilsson, Anton Nilsson, Kasper P Kepp, Jonas Björk, Stein Emil Vollset, Tea Lallukka, Ann Kristin Skrindo Knudsen

**Affiliations:** Department of Disease Burden, Norwegian Institute of Public Health, Bergen, Norway; Department of Disease Burden, Norwegian Institute of Public Health, Bergen, Norway; General Practice Research Unit (AFE) and Department of General Practice, Institute of Health and Society, University of Oslo, Oslo, Norway; Vårdcentralen Värmlands Nysäter and Centre for Clinical Research, County Council of Värmland, Varmland, Sweden; Department of Medical Epidemiology and Biostatistics, Karolinska Institute, Solna, Sweden; Unit of Epidemiology, Population Studies and Infrastructures (EPI@LUND), Division of Occupational and Environmental Medicine, Epidemiology, Lund University, Lund, Sweden; Section of Biophysical and Biomedicinal Chemistry, DTU Chemistry, Technical University of Denmark, Kongens Lyngby, Denmark; Epistudia, Bern, Switzerland; Unit of Epidemiology, Population Studies and Infrastructures (EPI@LUND), Division of Occupational and Environmental Medicine, Epidemiology, Lund University, Lund, Sweden; Clinical Studies Sweden, Forum South, Skåne University Hospital, Lund, Sweden; Institute for Health Metrics and Evaluation, University of Washington, Seattle, WA, USA; Department of Health Metrics Science, School of Medicine, University of Washington, Seattle, WA, USA; Department of Public Health, University of Helsinki, Helsinki, Finland; Department of Disease Burden, Norwegian Institute of Public Health, Bergen, Norway

## Abstract

**Background:**

The Nordic countries represent a unique case study for the COVID-19 pandemic due to socioeconomic and cultural similarities, high-quality comparable administrative register data and notable differences in mitigation policies during the pandemic. We aimed to compare weekly excess mortality in the Nordic countries across the three full pandemic years 2020–2022.

**Methods:**

Using data on weekly all-cause mortality from official administrative registers in Denmark, Finland, Norway and Sweden, we employed time series regression models to assess mortality developments within each pandemic year, with the period 2010–2019 used as reference period. We then compared excess mortality across the countries in 2020–2022, taking differences in population size and age- and sex-distribution into account. Results were age- and sex-standardized to the Danish population of 2020. Robustness was examined with a variety of sensitivity analyses.

**Results:**

While Sweden experienced excess mortality in 2020 [75 excess deaths per 100 000 population (95% prediction interval 29–122)], Denmark, Finland and Norway experienced excess mortality in 2022 [52 (14–90), 130 (83–177) and 88 (48–128), respectively]. Weekly death data reveal how mortality started to increase in mid-2021 in Denmark, Finland and Norway, and continued above the expected level through 2022.

**Conclusion:**

Although the Nordic countries experienced relatively low pandemic excess mortality, the impact and timing of excess mortality differed substantially. These estimates—arguably the most accurate available for any region in capturing pandemic-related excess deaths—may inform future research and policy regarding the complex mortality dynamics in times of a health crisis such as the COVID-19 pandemic.

## Introduction

The COVID-19 pandemic was characterized by atypical and complex mortality waves varying in strength, length and timing between countries. An in-depth investigation of the pandemic’s impact in comparable countries remains a crucial scientific inquiry that requires the best possible data and models. Excess mortality estimates the total mortality impact of the pandemic, including its likely effect on other causes of death,^1^ irrespective of variations in how countries report COVID-19 deaths.^2^^,^^3^ Numerous estimates of excess mortality during the first two years (2020 and 2021) have been published for different parts of the world,^4–9^ including the Nordic countries.^10–14^ However, these have not encompassed the full pandemic year of 2022 during which most restrictions were lifted, and the SARS-CoV-2 virus spread unrestrained. While international databases continually monitor excess mortality and offer tools and results for cross-country comparisons based on weekly data,^15^^,^^16^ they do not adjust for demographic changes occurring over time and across different countries. Accounting for demographic changes by standardizing populations with respect to narrow age bands and accounting for trends over longer reference time periods is however important for reliable estimates of expected deaths.^10^^,^^17^

The Nordic countries represent a unique case study of pandemic impact on mortality, due to their comparable high-quality data infrastructure, their geographical and socioeconomic similarities, and the notable variations in some mitigation policies during the pandemic.^18–20^ In Sweden, restrictions primarily relied on voluntary measures and recommendations, alongside limitations on the size of public gatherings, prohibition of visits to the elderly and the implementation of distance learning for individuals aged 17 years and older.^21^ Denmark, Finland and Norway, in contrast, opted for more stringent lockdown measures with closing of schools, kindergartens, work places and many other services.^18–20^ All the Nordic countries experienced relatively low excess mortality during the COVID-19 pandemic compared with most other countries in the world.^22^^,^^23^ This has been explained by effective public health measures, high trust in public institutions, well-functioning health-care systems and high vaccine uptake.^23^^,^^24^ However, mortality patterns varied between countries, with high excess mortality in Sweden in 2020 relative to the pre-pandemic period (2010–2019), but low excess mortality in 2021, compared with its neighbours.^10^^,^^11^ Yet, the COVID-19 pandemic, marked by atypical mortality waves, likely led to variations in the size and duration of mortality peaks within individual years. Previous studies that compared excess mortality across Denmark, Finland, Norway and Sweden and adjusted for demographic change have utilized yearly data, making corresponding analyses of within-year mortality variations unavailable.^10^^,^^11^ However, a precise evaluation of the impact of the pandemic on mortality requires not only adjustment for demographic changes, but also the use of high-frequency data and methodology that considers changes in both long-term and seasonal trends in mortality over time.^25^

To accurately understand the dynamics of pandemic mortality, we employed time series regression models to estimate and compare weekly excess mortality in Denmark, Finland, Norway and Sweden throughout the pandemic period 2020–2022. The analysis accounted for population age and sex distributions, carefully validated with sensitivity analysis.

## Methods

### Data

Population-based register data on number of deaths among country residents per week by age groups and sex in 2007–2022, as well as information about population count on either 31 December or 1 January each year were gathered from the official statistics released by the statistical authorities in Denmark, Finland and Norway (per October 2023). Data are of high quality and comparable across countries. For Sweden, the week of death was sometimes missing (1.8% of all deaths in 2010–2022). Therefore, we used data on weekly number of deaths sent to us directly from Statistics Sweden on 2 May 2023, which included deaths with unknown week. We then redistributed these to specific weeks using proportional redistribution based on the age- and sex-distribution of deaths within the same month (if month was known, 91% of the cases) or year (if month was not known, 9% of the cases). For each year, the population was set equal to the mean of the population count at the beginning and end of that year. Iceland was not included in the analyses due to small population with few weekly deaths, which makes estimation very uncertain for this country.

### Time series models

For each country, we used time series models to generate estimates of the expected age- and sex-standardized weekly mortality rate per 100 000 population with 95% prediction intervals for the pandemic period 2020–2022, with the years 2010–2019 as the pre-pandemic reference period. To enable comparisons, a common age- and sex-standardization was used for all countries based on the population in Denmark in 2020 with age groups 0–59, 60–69, 70–79, 80–89 and 90+. We included three time series models suitable for predicting weekly data to estimate the expected mortality rate; AutoRegressive Integrated Moving Average (ARIMA), Seasonal and Trend decomposition using Loess & innovations state space models for exponential smoothing (STL-ETS), and a Trigonometric exponential smoothing state space model with Box–Cox transformation, ARMA errors, Trend and Seasonal components (TBATS).^26^^,^^27^ In all models, we used a Box–Cox transformation to account for changes in seasonal variation over time. For each approach the model parameters (including the Box–Cox transformation parameter *λ*) were chosen based on minimizing the value of the Akaike information criterion (AIC) (separately for each country). All models were run using the ‘forecast’ package in R 4.2.1. The model specification for the individual models used in the ensemble is given in Supplementary table S1.

### Evaluation of model performance

To evaluate the performance of each time series model, we tested the fit between modelled and observed rates in the pre-pandemic period 2007–2019 for each model, in each country (for a more detailed description, see Supplementary material, page 1). Model fit was measured using mean absolute percentage error (MAPE) and results presented in Supplementary table S1.

### Final model applied

For the three time series models included (ARIMA, STL-ETS and TBATS), the fit between observed and expected mortality in 2007–2019 varied over time. As a result, we generated an ensemble model (three-model ensemble), in which the estimates from the ARIMA, STL-ETS and TBATS models were equally weighted. This was done separately for each country. The ensemble model constituted the final model used to estimate the expected weekly mortality rate in each country. The ensemble model was validated in the same way as each separate time series regression model (as explained above). A 95% prediction interval for the expected weekly rate was computed using Monte-Carlo simulation in which we resampled the weekly MAPE from the validation 10 000 times. The final lower and upper limit of the interval was based on the mean value of this MCMC simulation.

### Calculating excess deaths

Based on the expected weekly mortality rate from the final model, we calculated excess mortality per 100 000 as the difference in observed and expected age- and sex-standardized mortality rate, either weekly or annually (the latter found by summarizing over all weekly observed and expected mortality rates and calculating the difference).

### Sensitivity analyses

To assess how length of baseline affected the results we also performed sensitivity analyses, in which we ran the same analyses as in the main analysis but using a shorter reference period of five years (2010–2015). In addition, we ran the same linear regression models as published in a previous publication for the years 2020–2021,^10^ with the same age- and sex-standardization as in the main analyses but using annual instead of weekly data. The linear regression model predicts the annual standardized rate in the years 2020–2022 by extrapolating a linear trend line based on the years 2010–2019. In both sensitivity analyses, results were standardized to the Danish 2020 population.

## Results

**Figure 1:**
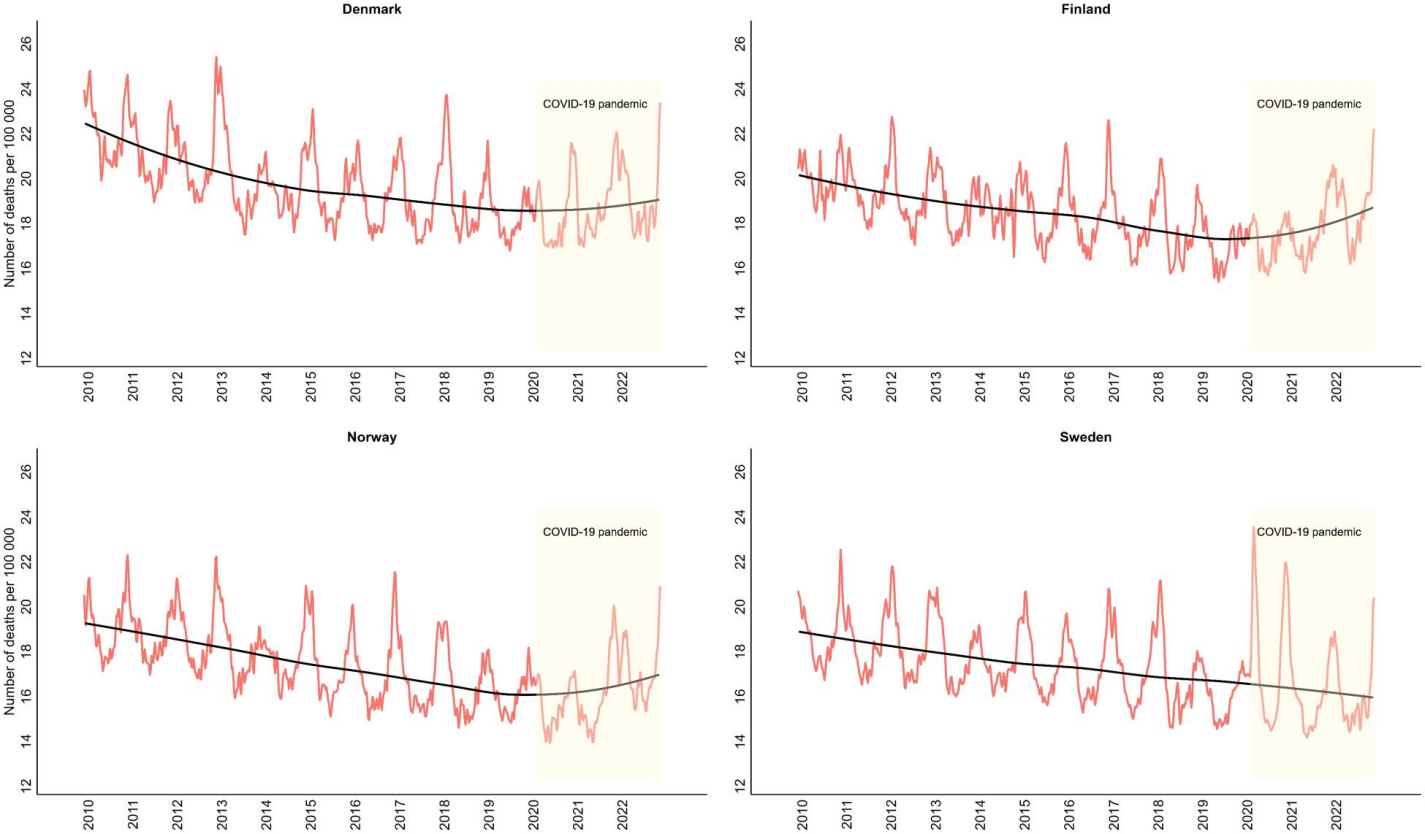
Weekly observed number of deaths (per 100 000) with a trend line (solid line using loess smoothing), using the Danish 2020 population as standard population (age- and sex-standardized), 2010–2022, by country

Prior to the COVID-19 pandemic, all four countries experienced a long-term downward trend in the mortality rate when accounting for changes in age- and sex distributions (figure 1). There were clear seasonal patterns in the mortality rate with peaks of mortality in the wintertime of varying height and length across years and countries. Denmark and Finland had higher levels of mortality compared with Norway and Sweden, which was reflected in higher weekly expected mortality rates during the pandemic years (Supplementary figure S1).

**Figure 2:**
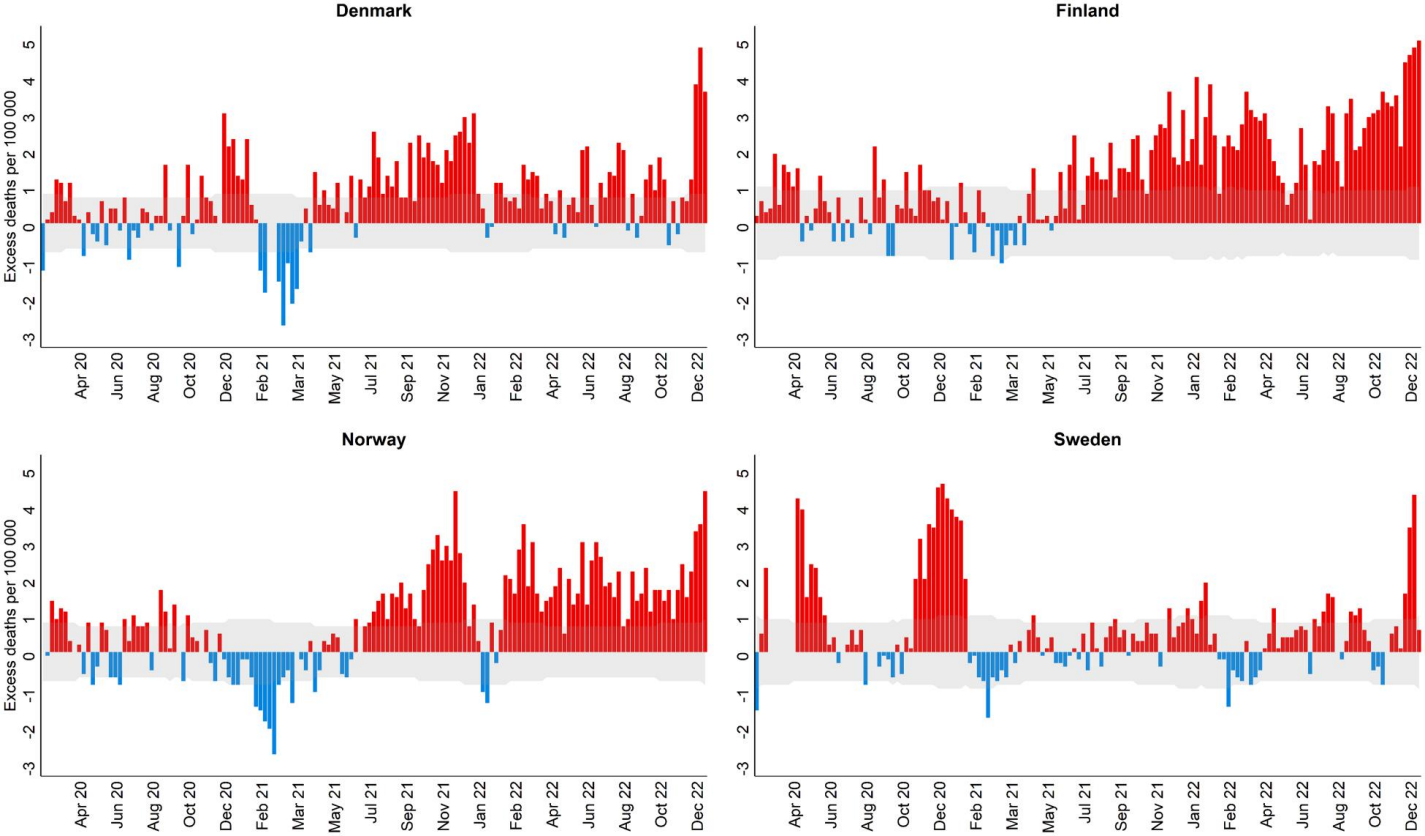
Weekly number of excess deaths (per 100 000) (with 95% prediction interval) in the pandemic period, weeks 11–52, by country, using the Danish 2020 population as standard population (age- and sex-standardized). Positive values indicate higher than expected weekly mortality, while negative values indicate lower than expected weekly mortality

Figure 2 shows the weekly number of excess deaths per 100 000 (age- and sex-standardized excess mortality rate) in each country during the pandemic years from our three-model ensemble (final model). While Sweden encountered large peaks in mortality during April–May and November–December 2020, Denmark, Finland and Norway, experienced very little excess mortality in this period. In the first half of 2021, all four countries experienced only few weeks with excess mortality, and in Denmark and Norway, in particular, there were several weeks with lower mortality than expected in the winter of 2020/2021. This was followed by prolonged periods of excess mortality commencing in summer 2021 and continuing throughout 2022 for Denmark, Finland and Norway (figure 2, Supplementary figure S1).

**Figure 3:**
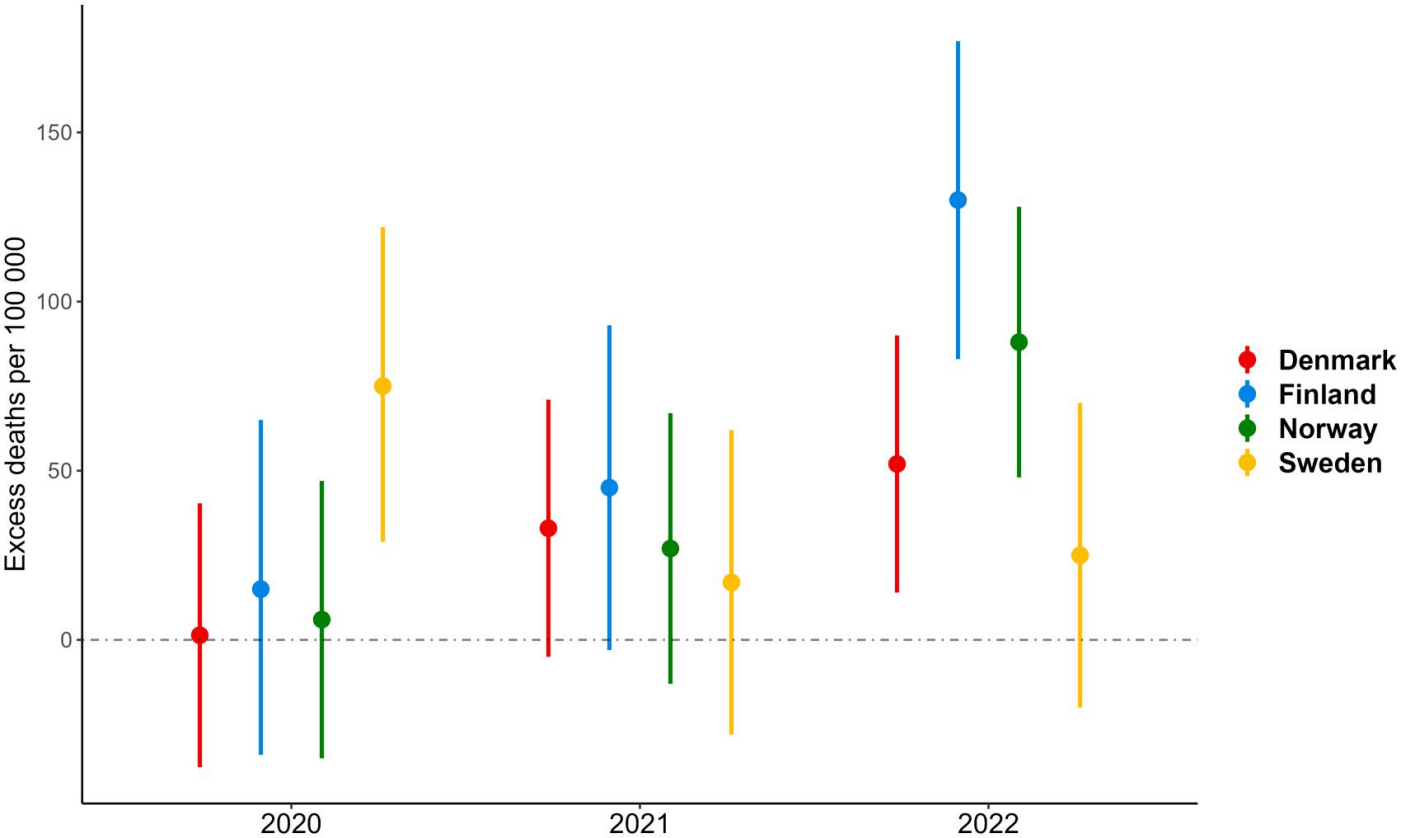
Annual number of excess deaths (per 100 000) (with 95% prediction interval) by country and year, using the Danish 2020 population as standard population (age- and sex-standardized)

Table 1 and figure 3 show the total annual number of excess deaths per 100 000 (excess mortality) when summing over all weeks in each year with results from our three-model ensemble. The estimates for the individual models used in the ensemble are given in Supplementary table S2. The elevated mortality rates observed in Sweden during 2020 led to 75 excess deaths per 100 000 individuals this year (95% prediction interval 29–122) (Table 1). The excess mortality depicted in the weekly data for Denmark, Norway and Finland in the second half 2021 did not manifest itself in any substantial or statistically significant numbers of excess deaths in the annual results. In 2022, however, substantial and significant excess mortality was seen in these three countries: 52 excess deaths per 100 000 for Denmark (95% prediction interval 14–90), 130 for Finland (83–177) and 88 for Norway (48–128). Aggregating over the three-year period (2020–2022) yielded excess deaths rates of 86 (−17 to 190), 190 (59–321), 121 (0–242) and 117 per 100 000 (−6 to 240) for Denmark, Finland, Norway and Sweden, respectively.

**Table 1:** Total number of observed deaths, crude number of deaths per 100 000 and age- and sex-standardized observed, expected and excess deaths per 100 000 using the Danish 2020 population as standard population

	Denmark	Finland	Norway	Sweden
Total observed^a^				
2020	55 478	56 237	41 207	99 613
2021	57 043	57 578	41 932	91 517
2022	59 277	63 046	45 583	94 160
Observed crude rate				
2020	951	1017	766	962
2021	974	1039	775	879
2022	1004	1135	835	898
Observed age- and sex-standardized rate				
2020	951	898	818	900
2021	960	897	816	818
2022	978	966	870	823
Expected age- and sex-standardized rate				
2020	950 (911 to 989)	883 (833 to 932)	812 (771 to 853)	825 (778 to 871)
2021	927 (889 to 965)	852 (804 to 900)	789 (749 to 829)	801 (756 to 846)
2022	926 (888 to 964)	836 (789 to 883)	782 (742 to 822)	798 (753 to 843)
*Excess age- and sex-standardized rate*				
2020	1 (−38 to 40)	15 (−34 to 65)	6 (−35 to 47)	75 (29 to 122)
2021	33 (−5 to 71)	45 (−3 to 93)	27 (−13 to 67)	17 (−28 to 62)
2022	52 (14 to 90)	130 (83 to 177)	88 (48 to 128)	25 (−20 to 70)
2020–2022	86 (−17 to 190)	190 (59 to 321)	121 (0 to 242)	117 (−6 to 240)

a Not age- and sex-standardized. Yearly numbers of observed deaths are obtained by summing weekly number of deaths and therefore do not fully correspond with the official number of annual deaths as reported by the statistical agencies, as the 52 (or for 2020, 53) weeks do not exactly correspond to calendar years. For Sweden, the observed number of deaths is based on data in which deaths with unknown week of death are redistributed to weeks within a year.

### Sensitivity analyses

When using the 5-years reference period of 2015–2019, estimates of expected mortality rates were higher for all four countries, resulting in lower estimates of excess mortality (Supplementary table S3). Sensitivity analyses based on yearly data using a linear model gave similar estimates for excess deaths as in the main analysis, but higher excess mortality for Denmark in 2022 compared with the weekly ensemble model (Supplementary table S4). Hence, numbers were largely comparable for all countries except Denmark (for 2022).

## Discussion

This study sought to estimate and compare excess mortality in Denmark, Finland, Norway and Sweden through the pandemic years 2020–2022 by using weekly data and a common age- and sex-standardization. Compared with the pre-pandemic period, Sweden experienced high excess mortality in 2020, a year with little or no excess mortality in Denmark, Finland and Norway. The latter three countries instead exhibited highest excess mortality in 2022. The findings of little excess mortality in 2021 in Norway, Denmark and Finland are nuanced by the observation of under-mortality in the first half, followed by an excess mortality in the second half of that year that continued throughout most of 2022.

The Nordic countries experienced low excess mortality in 2020–2022 compared with most other countries globally,^15^^,^^16^ but with significant variations between the Nordic countries in the timing of periods with excess mortality. Our results are in line with previous reports of high excess mortality in Sweden in 2020, and lower in 2021, and the opposite for Denmark, Finland and Norway, compared with pre-pandemic trends.^10^^,^^11^^,^^13^ The results for Norway and Sweden also correspond with a recently published paper, comparing mortality in these two countries during 2020–2022, that only included data for the first 43 weeks of 2022 and utilized a simpler methodological approach.^12^ By employing weekly data and including all three pandemic years 2020–2022, our results expand on these previous findings and show that excess mortality did not only differ between the years during the pandemic, but also by weeks within those years.

The neighbouring Nordic countries share many common aspects in terms of culture, lifestyle, economy, welfare and health-care organization. However, there was also differences between the Nordic countries before the pandemic, with lower life expectancy and higher disease burden in Denmark and Finland compared with Norway and Sweden.^28^ The four countries adopted different mitigation policies initially in the pandemic, providing an interesting case for comparison; while countermeasures were relatively similar, the strength of these, and the time points they were introduced, differed. Denmark, Finland and Norway had mandatory restrictions incorporated as new laws and regulations from the very beginning (March 2020).^18^^,^^19^ The measures in Sweden were more based on voluntary recommendations throughout the pandemic. This strategy was by many considered controversial and has been intensely debated.^21^^,^^29^ Total vaccine uptake was high and similar between the four countries, but vaccination roll-out differed somewhat, in which, e.g. Finland and Norway were behind the other Nordic countries in administrating the second dose,^24^ which could partly explain the observed variation in peaks of mortality.

Although analyses of potential reasons for the country-wise differences in mortality each pandemic year is beyond the scope of this article, one could speculate that different responses may have had an impact.^20^ Furthermore, the estimates may have been influenced by a mortality displacement, attributed to abnormally low pre-pandemic mortality in Sweden in 2019^30^ and the rigorous countermeasures in the other three countries, potentially contributing to enhanced longevity among frail individuals.^13^ We found signs of this in Denmark and Norway in which mortality rates were lower than expected in the winter of 2020/2021, followed by excess mortality from the latter half of 2021. Roughly twice as many were infected by SARS-CoV-2 in Sweden by November 2021 compared with its neighbouring countries.^31^ In 2020, excess mortality was mainly driven by COVID-19 associated deaths in Sweden,^13^^,^^32^ while mortality rates returned to more normal levels in 2021 and 2022, likely partly explained by a reduction in the population susceptible to mortality (mortality displacement) and higher immunity in the population. In Denmark, Finland and Norway, mortality started to increase in the fall of 2021 following the spread of the Delta variant, and later, the Omicron variant. These variants were more contagious, and the former one also caused more severe cases of disease than the Alpha variant.^33^^,^^34^ In late January/early February 2022, Denmark, Finland, Norway and Sweden lifted all restrictions. Following this, a large percentage of the population in the former three countries were infected,^35^^,^^36^ and there was excess mortality for most of this year.

Finally, both the pandemic in itself, and the responses, may have affected the incidence and mortality of other diseases and health outcomes.^32^^,^^37^ In a parallel work, we report excess mortality due to cardiovascular diseases and under-mortality due to respiratory diseases other than COVID-19 during 2020–2022 in Denmark, Finland, Norway and Sweden.^38^ These results suggest that several mechanisms that affects excess mortality have been at play in this period.

### Methodological considerations and limitations

Estimates of excess mortality will vary according to inputs and methods used.^39^ Consequently, we performed several sensitivity analyses, and, on the whole, these yielded results consistent with the main analyses strengthening the conclusions of the article. One important consideration when estimating expected mortality is which years to include in the reference period. Many previous studies have chosen the reference period 2015–2019.^5^^,^^6^ In the present study, utilizing a 5-year reference period (2015–2019) resulted in higher expected mortality rates than when using a 10-year reference period, and hence lower estimates of excess mortality, in all four countries in the three-model ensemble, but to a varying degree. The latter can likely be attributed to the extent to which each country experienced unusual mortality events in the years prior to the pandemic, as shown in previous work.^10^ For instance, Denmark saw a spike in mortality during the severe influenza season of 2017/2018, while both Denmark and Sweden observed unusually low mortality in 2019. By adding a longer reference period, we expected to pick up the underlying trend more reliably in mortality, not impacted by specific recent unusual mortality events.

We used a three-model ensemble to estimate the expected age- and sex-standardized mortality rate. In general, TBATS produced higher estimates (hence gave lower excess mortality) by putting more weight on trends in recent years before the pandemic compared with ARIMA and STL-ETS. The results from each model were weighted equally in the models as their performance varied over time. A different weighting would give slightly different estimates but would not alter our main conclusions. Using the pre-pandemic period 2010–2019 as a reference for the pandemic years 2020–2022, we predicted weekly death counts up to 157 weeks, resulting in increased within-model uncertainty over time.

Using a linear model with annual data and 2010–2019 assumes that each age group follows a linear trend in mortality throughout the whole period 2010–2022. The estimates for excess deaths were similar as with the three-model ensemble model, except for Denmark, in which the linear model estimated higher excess mortality for 2022. This may be due to a combination of poorer fit of the linear regression model for Denmark, and the ensemble model assigning greater significance to more recent mortality trends.

The Nordic countries constitute a unique case study for exploring pandemic impact on mortality, sharing many demographic characteristics and access to comparable high-quality health register data. Although these four countries are similar in many respects, marked differences in excess mortality during 2020–2022 were noticed in our work, both within and across years. While Sweden experienced large peaks in mortality in 2020, Denmark, Finland and Norway had longer periods of excess mortality from the latter half of 2021. Our findings warrant a comprehensive investigation of cause-of-death mortality, as well as continued assessment of post-pandemic mortality trends. This is crucial to gain a better understanding of the long-term effects of the COVID-19 pandemic and to develop effective strategies to respond to future epidemics and pandemics.

## Supplementary Material

ckae091_Supplementary_Data

## Data Availability

All data required for the calculations in this work are available at the web pages of Statistics Norway, Statistics Denmark and Statistics Finland, except for data on weekly deaths by age in Sweden which were prepared and sent to us directly by Statistics Sweden. Norway: https://www.ssb.no/en/statbank/table/12954/ (weekly deaths) https://www.ssb.no/en/statbank/table/07459/ (population). Sweden: https://www.statistikdatabasen.scb.se/pxweb/en/ssd/START__BE__BE0101__BE0101A/BefolkningNy/ (population). Denmark: https://m.statbank.dk/TableInfo/DODC2 (weekly deaths) https://www.statbank.dk/BEFOLK1 (population). Finland: https://pxdata.stat.fi/PxWeb/pxweb/en/Kokeelliset_tilastot/Kokeelliset_tilastot__vamuu_koke/koeti_vamuu_pxt_12ng.px/ (weekly deaths) https://statfin.stat.fi/PxWeb/pxweb/en/StatFin/StatFin__vaerak/statfin_vaerak_pxt_11rd.px/ (population). All scripts used in the present study are available upon request by contacting the corresponding author. Key points An evaluation of excess mortality in the Nordic countries during the COVID-19 pandemic is of great interest, given these countries’ similarities and diverse mitigation policies. An accurate evaluation requires use of data and methods that adjust for demographic changes and accounts for long-term and seasonal mortality trends over time. There were distinct variations in excess mortality across and within years, with pronounced peaks of excess mortality in Sweden in 2020, while Denmark, Finland and Norway exhibited prolonged periods of increased mortality from the latter half of 2021, persisting throughout much of 2022. The findings underscore the need for in-depth explorations of the impact of the pandemic and the complex dynamics contributing to divergent mortality patterns in otherwise comparable countries.
