# Supplementary material for: Excess mortality in Denmark, Finland, Norway and Sweden during the COVID-19 pandemic 2020–2022
Source: Eur J Public Health. 2024 May 17;34(4):737–43. doi: 10.1093/eurpub/ckae091 (PMC11293825; doi:10.1093/eurpub/ckae091)
Supplement: ckae091_Supplementary_Data [file ckae091_supplementary_data.pdf]

Supplementary material

Description of validation

To evaluate how well each model performed, we compared the accuracy of each model using data from 2007–2019 for each country. Accuracy was evaluated by calculating the mean absolute percentage error (MAPE), which measures the mean percentage difference between the observed and predicted value. The models were validated using data from 2007–2019. The data sets used in the validation were the same as when predicting death rates for 2020–2022 in each country. The only exception was Sweden, in which the data set with information on number of deaths with unknown week only contained data for the period 2010–2019. Therefore, for the validation, we used data from Statistics Sweden on weekly deaths (2007–2019) sent to us directly by Statistics Sweden the 26th of September 2023. In this data set, deaths with unknown week of death were not included.

In the validation, we divided the data for the period 2007–2019 into three training data sets of 10 years (2007–2016, 2008–2017, 2009–2018) and three test data sets of three, two and one year (2017–2019, 2018–2019 and 2019). In each validation set, the mean absolute percentage error (MAPE) was calculated and used to evaluate the accuracy of each model.

Supplementary tables

Supplementary table 1: Model specification[a] for each model (with mean absolute percentage error (MAPE)[b]) used to estimate expected mortality and included in the three-model ensemble, by country.

**Model**
**Denmark**
ARIMA – Autoregressive moving average (1,0,1) (1,1,0) [52] with drift (MAPE=4,62)<br>STL-ETS(A,N,N) – Seasonal and Trend decomposition using Loess & innovations state space models for exponential smoothing (simple exponential smoothing with multiplicative errors) (MAPE= 4,44)<br>TBATS – Trigonometric exponential smoothing state space model with Box–Cox transformation, ARMA errors, trend and seasonal components (0.217, {0,0}, 0.8, {<52.18,5}) (MAPE=4,20)
**Finland**
ARIMA – Autoregressive moving average (2,1,1)(1,0,0)[52] (MAPE=4,88)<br>STL-ETS(M,N,N) – Seasonal and Trend decomposition using Loess & innovations state space models for exponential smoothing (simple exponential smoothing with multiplicative errors) (MAPE=5,96)<br>TBATS – Trigonometric exponential smoothing state space model with Box–Cox transformation, ARMA errors, trend and seasonal components (1, {0,0}, 0.8, {>52.18,5>}) (MAPE=5,55)
**Norway**
ARIMA – Autoregressive moving average (1,0,2)(1,1,0)[52] with drift (MAPE=5,23)<br>STL-ETS(A,N,N) – Seasonal and Trend decomposition using Loess & innovations state space models for exponential smoothing (simple exponential smoothing with additive errors) (MAPE=6,29)<br>TBATS – Trigonometric exponential smoothing state space model with Box–Cox transformation, ARMA errors, trend and seasonal components TBATS(1,{0,0}, 0.844, {<52.18,6>}) (MAPE=4,58)
**Sweden**
ARIMA – Autoregressive moving average (1,0,1)(1,1,0)[52] with drift (MAPE=4,80)<br>STL-ETS(A,N,N) – Seasonal and Trend decomposition using Loess & innovations state space models for exponential smoothing (simple exponential smoothing with additive errors) (MAPE=5,99)<br>TBATS – Trigonometric exponential smoothing state space model with Box–Cox transformation, ARMA errors, trend and seasonal components (0.884, {0,0}, 0.8, {<52.18,6>}) (MAPE=5,84)

[a]See Hyndman RJ, Athanasopoulos G. Forecasting: principles and practice 2nd ed. Melbourne, Australia OTexts 2018 for description of model parameters. [b]MAPE is the mean of MAPE across all three evaluations of model fit (validation). First, MAPE is calculated for every week in the test set, then mean MAPE is calculated across all weeks. This results in three values for MAPE, one for each of the three test data sets. In the next step, the average is taken across the three values.

Supplementary table 2: Age- and sex-standardised expected mortality rate per 100,000, and excess number of deaths per 100,000 by country and year from each model included in the three-model ensemble, using the Danish 2020 population as standard population.

	Denmark	Finland	Norway	Sweden
**Expected rate**				
ARIMA				
2020	952 (907–998)	900 (854–945)	817 (772–862)	832 (794–870)
2021	910 (867–953)	850 (807–893)	782 (739–825)	792 (756–828)
2022	895 (853–938)	811 (770–852)	762 (720–804)	782 (746–818)
STL-ETS				
2020	940 (897–983)	844 (784–904)	792 (739–846)	811 (757–865)
2021	925 (882–967)	818 (760–876)	773 (721–825)	794 (741–847)
2022	929 (886–971)	809 (752–867)	771 (720–823)	794 (741–848)
TBATS				
2020	957 (913–1001)	904 (849–959)	826 (781–870)	831 (775–887)
2021	947 (904–991)	887 (833–941)	812 (768–856)	816 (761–871)
2022	954 (910–998)	887 (833–941)	812 (768–856)	817 (762–872)
**Excess rate**				
ARIMA				
2020	-1 (-47–44)	-2 (-47–44)	1 (-44–46)	68 (30–106)
2021	50 (7–93)	47 (4–90)	34 (-9–77)	26 (-10–62)
2022	83 (40–125)	155 (114–196)	108 (66–150)	41 (5–77)
STL-ETS				
2020	11 (-32–54)	54 (-6–114)	26 (-28–79)	89 (35–143)
2021	35 (-7–78)	79 (21–137)	43 (-9–95)	24 (-29–77)
2022	49 (7–92)	157 (99–214)	99 (47–150)	29 (-25–82)
TBATS				
2020	-6 (-50–38)	-6 (-61–49)	-8 (-52–37)	69 (13–125)
2021	13 (-31–56)	10 (-44–64)	4 (-40–48)	2 (-53–57)
2022	24 (-20–68)	79 (25–133)	58 (14–102)	6 (-49–61)

ARIMA: Autoregressive moving average, STL-ETS – Seasonal and Trend decomposition using Loess & innovations state space models for exponential smoothing, TBATS – Trigonometric exponential smoothing state space model with Box–Cox transformation, ARMA errors, trend and seasonal components.

Supplementary table 3: Age- and sex-standardised expected mortality rate per 100,000, and excess number of deaths per 100,000 using our ensemble model with a 5-year reference period (2015-2019), by country and year, using the Danish 2020 population as standard population.

	**Denmark**	**Finland**	**Norway**	**Sweden**
**Expected rate**				
2020	962 (919–1004)	894 (836–952)	826 (785–867)	826 (773–879)
2021	939 (898–981)	873 (817–930)	805 (765–845)	820 (768–873)
2022	940 (899–981)	873 (817–929)	802 (762–842)	853 (798–908)
**Excess rate**				
2020	-11 (-53–32)	4 (-54–62)	-8 (-49–33)	74 (21–127)
2021	21 (-21–62)	24 (-33–80)	11 (-29–51)	-2 (-55–50)
2022	38 (-3–79)	93 (37–149)	68 (28–108)	-30 (-85–25)

Supplementary table 4: Excess number of deaths 100,000 by country and year from linear models with annual data, using the Danish 2020 population as standard population (age- and sex-standardised).

	**Denmark**	**Finland**	**Norway**	**Sweden**
**Expected rate**				
2020	21	8	3	65
2021	65	36	33	14
2022	103	121	115	34
2020-2022	189	164	151	113

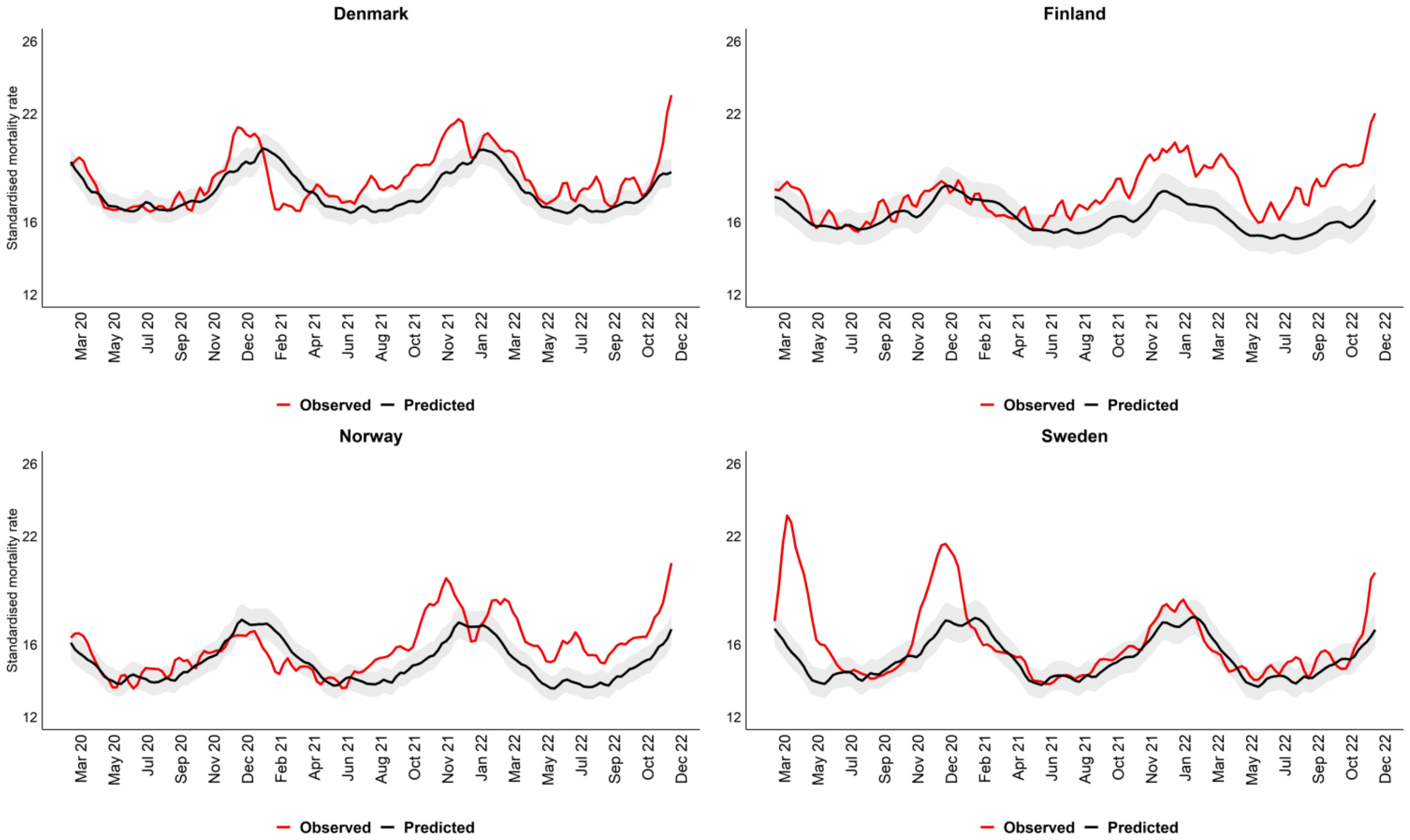

Supplementary figure 1: Weekly number of age-standardised observed (in red) and expected number of deaths (in black) per 100,000 with 95% prediction interval by country, using the Danish 2020 population as standard population.
